# The Advancement of Dental Science

**Published:** 1873-03

**Authors:** H. M. Grant


### AMERICAN JOURNAL

OF

### DENTAL SCIENCE.

Vol. VI. THIRD SERIES--MARCH, 1873. No. 11.

### ARTICLE I.

### *The Advancement of Dental Science.*

An Address delivered before the Virginia State Dental Association.

BY H. M. GRANT, D. D. S., M. D.

*Gentlemen of the Virginia State Dental Association:*

The contemplated objects of this Association, when organized, as set forth in the preamble of its constitution, are "to cultivate the science of Dentistry, and all its collateral branches; to elevate and sustain the professional character of the members of the profession, and to promote amongst them mutual improvement, social intercourse, and good will." And we would here further add: The general advancement of Dental Science throughout the good old State of Virginia.

The accomplishment of such desirable ends, we must all admit, would be an achievement of which we should all be proud indeed; and which should stimulate each and every one of us to do his whole duty. These objects, if accomplished, must result in great good to the dental profession, as well as of inestimable value to our patrons—who, from day to day, week to week, and year to year, place themselves under our hands for relief and treatment, and trust themselves to our skill. It then becomes our duty, individually and collectively, honestly and earnestly, to devise the ways and means best calculated to fit ourselves for the faithful discharge of the weighty and responsible obligations which rest upon us. Nor should we relax our diligence while there yet remains a field of knowledge unexplored, and until every hidden truth shall have been brought to light. Not until the microscope, the scalpel, and the crucible have no more to reveal, and all the fields of professional enquiry have been exhausted, and their fruits reaped, and their harvest garnered.

It becomes my duty, as the presiding officer of this Association, to point out to you some of the ways and means suggested to my mind by my past experience, and from all I have been able to collate from the experience of others, best calculated to advance the science of dentistry in our beloved State.

The humiliating experience of our sister profession, Medicine—I should more properly say our mother profession—should teach us the folly of supinely waiting for legislation in our behalf, or seeking its co-operation. If obstacles in the way of establishing a sentiment in favor of dentistry in Virginia are to be overcome; if enemies are to be vanquished, and if the science of dentistry is to be made progressive, we, the dentists of Virginia, must put our hands to the plow, and look not back until our progression shall have been placed side by side with the noblest profession of the land—honored and respected by the wisest and best of society.

To accomplish a desideratum so important to our profession, so essential to the great cause of civilization and humanity, should surely be stimulus enough to inspire every member of this Association with a desire that his influence be felt and recognized. To this end I call on every member of this Association, every dentist, and every good citizen of Virginia, whose mind is not indifferent to scientific truths, and whose heart is not recreant to the best interests of his fellow-man, to come forward and lend us a helping hand; to give us his hearty co-operation; remembering that social union brings light, while discord alone brings darkness.

It matters not where the idea of organization or association originated—whether among the ancient Jews, as is generally conceded among minds of antiquarian research or in some of the younger forms of human government; yet it is now conceded on all hands that organization is essential to success in almost all the works of human life.

It is true that no man can lose his personal identity, nor transfer his responsibility to his fellow-man; his personality is not absorbed nor alienated by his incorporation into an organized body or association. But on the contrary, just as it requires the combination of organs composed of bones, muscles, blood, and nerve force to complete the human system, so does it require a combination of individual members to complete a perfect organization competent for the discharge of its legitimate duties. And as in the human body, the thinking mind will give direction to all members, and exercise control upon all the organs, so as to regulate the movements of all to the furtherance of the general good, so in the workings of these corporate bodies or associations, it is well to have a head, responsible and efficient, to regulate and control the movements of the rest. And as the heart will send the life current rushing through every artery and vein, so will this throbbing organism send the currents of energy and vitality pulsating through all the members that composes the arteries and veins of this organic associated system.

It is upon these fundamental principles of what I conceive to be a sound philosophy that our Virginia State Dental Association is formed. Every member is a component element in this organized body; he preserves his own identity, his own personality—for be it remembered, this is not a chemical compound, but rather a mechanical mixture—where each ingredient retains its normal characters, while in a chemical compound the elements form an entirely different substance.

Where there is a mechanical mixture, there is usually a preservation of the original ingredients, and yet a change is produced by the combination of the different elements. The individual dentist, then, has as distinct a personality as he ever had before; but by being associated into a corporate body, he becomes an important element in what is emphatically a new body—just as the individual capitalist may retain his own ideas of finance, even when he becomes a director or a stockholder in a bank; just as every citizen may have and retain his individual opinions and predilections when he becomes a member of the Legislature.

But the combination of citizens duly elected constitutes the Legislature of a State; a body of capitalists voluntarily associated constitutes a banking company; and on the same principle, and for mutual benefit and protection, we have Boards of Trade, Chambers of Commerce, Agricultural Societies, &c.

In all of these, certain rights and privileges are surrendered for the common good, and the general purposes of trade, commerce and agriculture.

These gentlemen will combine all their energies and activities for their mutual benefits. And now be it remembered, in all such combinations, when properly regulated and economically managed, there is greater power, greater efficiency, greater usefulness, from the combination, than the mere advantage of each separate energy and efficiency.

Men thus organized are a direct contradiction to the old problem which is true in physics, but false in metaphysics: "A whole is equal to the sum of all its parts." On the contrary, in subtle fields of metaphysics, the whole is always greater than the sum of all its parts. Mind rubbing against mind will produce friction; and this will generate the electric spark of genius, science and wisdom. The combination of mental energies is not like the combination of muscular energies.

The sum of the power of twelve muscular men is only twelve times the power of one man. But the sum of the mental energies of twelve thinking men is like the application of fire to the steam engine—that by turning water into steam will multiply the power beyond all computation.

And now let us look at the practical results of these principles in their application to Dental Science. Every dentist, in the course of his experience, must have presented to him some difficult cases, in which some peculiar phenomena in the case has baffled his skill; in other words, his judgment has deceived him, and the result of his labors is not what he expected or desired. He has not time to refer to his text books, and if he had, they are not at his hand. If he then has a professional brother to whom he can go for counsel, he can verify the old maxim: "That in the multitude of counsellors there is wisdom."

There are times that we must act immediately; we have no time to consult authority; cases wherein our sympathies are very much enlisted; when we would like to have the judgment of some one in whose skill we had confidence. Hence it is, that if our associations are formed, we can have the experience of the oldest and wisest to guide us in making up our diagnosis. Here we can discuss the mooted points in any given case; here we can report our failures as well as our successes; as well as subject to the test of reason and experience the value of every new thought, process or appliance offered to the dental world.

Again, every intelligent and right-minded member of the dental profession in Virginia must feel a contempt and disgust for those self constituted exotics that are springing up all over our land—who, without leave or license, are degrading our profession by their reckless experiments upon the teeth of the unsuspecting victims, who are unfortunately incompetent to discriminate between the true dentist and the quack. If we could bring to bear upon them all the power of our organization, we can produce a sentiment in the public mind that will compel these adventurers to either submit themselves to a proper course of training in some reputable dental office, and in some dental college, or sink them into their merited contempt and obloquy. If the public mind could be aroused and educated as it should be, our profession in Virginia will assume a very different status to what it now does. These and other sources of power must be tried in Virginia in order to overcome the many obstacles in the way of progress in dental science. Professional pride must be thoroughly aroused. The people must be taught to demand what is their just rights. The press, the power of which has never been tried in behalf of dental science, must be invoked, and the pen, which is second alone to the press, must be wielded until our profession is placed side by side with any of the other learned professions of the age.

Our profession in Virginia for years past has floated along like a deserted ship. It has been anxiously watched by some of its members. They have now organized themselves into a bold and manly crew, who, though small in number, have at last boarded her, and if the helmsman will stand firm at the wheel, they will sail her triumphantly amid the rough breakers, under cozy but sound canvas; and if those who take passage with us will do their duty, we will soon so extend our sphere of usefulness, as we pursue the journey of professional life, as to command the respect of those rich with intellectual culture, social and moral worth, and in fact the civilized world.

And now, gentlemen, if ever these few objects, among a multitude of others that I might mention, are to be obtained by a thorough organization, are they not worthy of all our self-denial, and all our labors, to obtain even them? And if ever this much be obtained, must they not conduce to the elevation of our noble profession? Surely no one who loves truth as it is revealed in science, or has a feeling of humanity, or a sentiment of sympathy for the suffering, can deny that our undertaking is, at least, worthy of the esteem of all. And in my humble opinion, no dentist who has a proper regard for his profession, can refuse to lend all his influence to the furtherance of our noble and patriotic organization. Then let us put our own shoulders to the wheel, and show to the world by our concert of action, unity of purpose, and persistent efforts, that we are in earnest; that we mean business; that we have the good of our fellow-beings at heart; that our motives are pure, laudable and patriotic.

In order that we may derive the full force of organization, all the parts of this organic machine should be well adapted to the general whole. The bearings of the machinery must be properly turned and oiled. We need prudence, zeal and integrity, to make this association what it should be; a bond that will unite our profession in friendship, a spur to the indolent, a beacon star to the studious, a crown to the good and true; but a whip of scorpions to the undeserving.

I therefore, respectfully suggest for your consideration, the following general plan: let each member of this body on his return to his home, his field of labor, endeavor to exercise his influence towards educating the public mind, as to the present status of our profession at present, and its arguments; educate them as to the demands of the present standard of dental education, teach them the difference between the true dentist and empiric, give them to understand that mere mechanical skill is not evidence of ability sufficient on the part of any one to become a successful dentist.

A good dentist can only be made out of the proper material; without which, neither collegiate nor private instructions can avail. He must be a man of talents, and of good artistic and mechanical tastes: a gem, however, rough, might be polished, and its beauties brought out; but if it is only a worthless stone, no amount of labor can impart the lustre which is latent in that, that it imitates, a jewel of the first water.

The public has its eye upon us; our actions as a body, and as individuals are closely watched; let us then follow out this idea, to say and do something every day of our lives, that will educate the communities in which we respectively live, so that they will appreciate us as men of taste, of letters, and of refinement. Let us endeavor to raise the standard of dental education. That the dental colleges have been remiss in their duty in not demanding a higher grade of qualification before graduating their students, I suppose none will now deny, but we have good reason to hope that ere long, such a condition of things will have passed away.

To remedy this evil, we should pluck the beam from our own eyes, and then take the mote from the eyes of the different dental colleges. I know dentists who take young men as assistants for a few weeks or months, and then turn them out to prey upon the public. Until the profession puts its veto upon such proceedings it will be overrun with impostors. Every dentist should reject all applicants for office instruction who will not obligate himself to devote at least two years to pupilage. For my own part I require two years private pupilage, and attendance upon full courses of lectures.

I would also suggest the propriety of this Association stimulating its members to write essays upon some subjects of interest to our profession; to report all facts and interesting cases; these papers to be carefully preserved by the Secretary, and read to the Association at its next annual meeting, whose right it shall be to preserve and publish all that shall be of sufficient interest. By so doing we would add to the sum of practical facts, as well as to elevate the standard of the dental literature of our State.

And now, gentlemen, I will not occupy your valuable time any longer on this occasion; but I would be recreant to the best emotions of my nature, and the impulses of a grateful heart, if I hesitated to acknowledge my sincere and warmest thanks to you for the distinguished honor you conferred on me in November last in electing me to preside over your deliberations. I assure you that no one was ever more sensible to my own short-comings, and incompetency to the task.

But in endeavoring to do my duty I have the consciousness of having been aided and sustained by you. It is my pleasure to say that I have received from you only that patient indulgence, that gentle and courteous return, as honorable to yourselves as it has been gratifying to me.

This being my experience with the character of those constituting the Virginia State Dental Association, I am left without a doubt as to the dignified and courteous manner with which its deliberations will be conducted.

I again thank you for your kindness and indulgence, with the hope that this Association may exist when those of us who now constitute its members shall have been gathered to our Father's; and that each one of you may live to witness your labors crowned with an honorable success, and your brows encircled with a halo of imperishable renown.

---

